# An alternate solution for the treatment of ascending aortic aneurysms: the wrapping technique

**DOI:** 10.1186/1749-8090-5-100

**Published:** 2010-11-03

**Authors:** Georgios I Tagarakis, Dimos Karangelis, Andony J Baddour, Marios E Daskalopoulos, Vassilios T Liouras, Dimitrios Papadopoulos, Konstantinos Stamoulis, Stefania S Lampoura, Nikolaos B Tsilimingas

**Affiliations:** 1 Department of Cardiovascular and Thoracic Surgery, University of Thessaly, Larissa, Greece; 2 Department of Anesthesiology, University of Thessaly, Larissa, Greece

## Abstract

**Background:**

The aortic Dacron wrapping technique is a surgical technique used under certain circumstances in cases of ascending aorta dilatation. Herein, we are presenting our experience on the method performed on multimorbid patients who denied major aortic surgery.

**Methods:**

We included in our series 7 patients (5 male-2 female) with mild to moderate ascending aortic dilatation, who were operated with the wrapping technique. One patient was submitted to biological aortic valve replacement during the same procedure. The number of conventionally operated patients during the same period (2 years) was 21.

**Results:**

Mortality during the 18-months follow-up control was 0%. One patient had to be operated with biological aortic valve replacement 18 months after the initial wrapping operation, although the diameter of her ascending aorta remained stable.

**Conclusions:**

The Dacron wrapping technique is a method that can alternatively be used in multimorbid patients with mild to moderate ascending aortic dilatation without dissecting elements and has generally good results.

## Letter to the Editor

### Dear Editor

Even in the current time of advanced surgical procedures and endovascular alternative techniques for the treatment of aortic pathologies, ascending aortic aneurysms remain a challenging problem for every cardiac surgeon. This is why our utmost interest was focused on the article recently published in your esteemed journal by Ang et al [1]; it dealt with the interesting topic of ascending aortic wrapping in cases of mild to moderate ascending aorta dilatation during aortic valve replacement procedures. Herein, we would like to present our own experience with the wrapping technique, which we used as an alternative procedure in the two-year period 2007 and 2008 in a series of 7 multimorbid patients who were at risk for major surgery, which they definitely refused. During the same period, the number of patients with ascending aortic aneurysm who were submitted to conventional ascending aortic aneurysm operation in our department was 21. The wrapping technique was not necessarily combined with aortic valve repair/replacement, and the morbid conditions of our patients included combinations of advanced age and severe organic or metabolic insufficiencies, such as diabetes mellitus, hypothyreoidism, coronary artery disease, previous CABG, severe heart or renal failure, chronic obstructive pulmonary disease and adipositas per magna. These patients, whose characteristics can be seen in table 1, concentrated classical indications for ascending aorta replacement, but, due to the increased surgical risk and their wish to avoid major surgery, they were submitted to the Dacron wrapping technique, with excellent results in the long-term (18 months ) follow-up control.

**Table 1:** Patients' baseline characteristics and comorbidities

Patient number	Gender	Age	Aortic valve insufficiency/indication for surgical repair or replacement	Ascending aortic diameter	Comorbidities
Patient 1	Male	74	Mild to moderate, aortic valve replacement performed	5.3 cm	Diabetes mellitus, arterial hypertension, renal failure,

Patient 2	Female	70	Mild	5.4 cm	Diabetes mellitus, arterial hypertension, adipositas per magna, heart failure (left ventricular ejection fraction 45%)

Patient 3	Male	78	Mild	5.3 cm	Diabetes mellitus, arterial hypertension, renal failure (dialysis), previous CABG

Patient 4	Male	74	Mild	5.5 cm	Diabetes mellitus, arterial hypertension, coronary artery disease, previous CABG

Patient 5	Male	80	Mild	5.4 cm	Diabetes mellitus, arterial hypertension, hyperlipidemia, coronary artery disease, heart failure, left ventricular ejection fraction 30%)

Patient 6	Female	73	Mild	5.4 cm	Arterial hypertension , chronic obstructive pulmonary disease, adipositas per magna

Patient 7	Male	77	Mild	5.4 cm	Arterial hypertension, previous CABG, chronic obstructive pulmonary disease

Intra-and perioperative mortality was 0%. In the 18 months' follow-up control none of the patients presented with augmented ascending aortic diameter (as measured per echocardiography and CT angiography). Patient No 2 developed progressive aortic valve insufficiency (with stable aortic diameter), and was submitted to successful biological aortic valve replacement 18 months after the initial operation.

In regard to the issue of neurological and neuropsychiatric complications (stroke, transient ischemic attacks, postoperative delirium), which can consist a major problem after aortic surgery, we are glad to report that no incidents of the kind were observed.

In conclusion, the Dacron wrapping technique can be an alternative solution for ascending aortic aneurysms without dissecting elements in cases of severely morbid patients who are unwilling to undergo major aortic surgery due to the significantly increased perioperative risk. We would however wish to emphasize that this technique should not be misused as a standard procedure in cases of ascending aortic dilatation, but adopted only in exceptional cases where mild to moderate dilatation, advanced age and major comorbidities are combined with the patient's wish to avoid major aortic surgery.

## Conflicts of interest

The authors declare that there are no conflicts of interest.

## Authors' contributions

G T is the main author of the manuscript and member of the surgical team. DK coauthored the paper. AB was a member of the surgical team. MD performed linguistic control. VL was a member of the surgical team. DP was member of the anesthesiological team. KS was member of the anesthesiological team. SL performed linguistic control. N T was the primary surgeon and performed the final control.
